# Enhancing open clinical trials through blinded evaluations: an exploration with diabetic foot infections

**DOI:** 10.1186/s13063-023-07652-y

**Published:** 2023-11-09

**Authors:** Qingna Li, Mengli Xiao, Xingfang Liu, Yang Zhao, Haoling Zhang, Yundong Yin, Panbo Qiu, Fang Lu, Rui Gao

**Affiliations:** 1 Institution of Clinical Pharmacology, Xiyuan Hospital of China Academy of Chinese Medical Sciences, Beijing, China; 2 National Clinical Research Center for Chinese Medicine Cardiology, Beijing, China; 3 Key Laboratory for Clinical Research and Evaluation of Traditional Chinese Medicine of National Medical Products Administration, Beijing, China; 4 Research Department, Swiss University of Traditional Chinese Medicine, Bad Zurzach, Switzerland

**Keywords:** Blinded evaluation, Open clinical trial, Diabetic foot infection

## Abstract

**Background:**

Blinding drugs through simulation techniques is an important means to control the subjective bias of investigators and subjects. However, clinical trials face significant challenges in the placebo production of drugs, and many trials cannot be double-blinded.

**Objective:**

This study was conducted to ascertain the consistency between non-blind and blind evaluation results in clinical trials and to pioneer strategies to control information bias, particularly in trials where double-blinding is not feasible.

**Methods:**

In this investigation, a randomized controlled trial (RCT) studying diabetic foot infections (DFIs) was utilized as a representative case. In this trial, the grading of DFIs, as per guidelines by the Infectious Disease Society of America (IDSA) and International Working Group on Diabetic Foot (IWGDF), was used as the primary efficacy indicator. A sample of sixteen patients was randomly chosen from the RCT, and DFI grading was assessed jointly by both non-blinded investigators and blinded center-reading investigators. A consistency test was then deployed to compare the evaluation results, forming the basis for our proposed strategies for effective blinded evaluation. In addition, other perspectives were collected at the end of this study, including with those involved in designing and conducting the recent blinded evaluation trial.

**Results:**

Five subjects were excluded due to the quality of photos or the lack of post-treatment visits. The post-treatment IDSA/IWGDF grading results were compared in 11 subjects (experimental group=6, control group=5), and the consistency test showed inconsistent results between the non-blinded and center reading blinded evaluations (Kappa=0.248, *p*=0.384). In the experimental group, three cases were judged as grade 1 in the non-blinded evaluation and grade 2 in the central reading blinded evaluation; in the control group, three cases were judged as grade 2 in the non-blinded evaluation and grade 1 in the central reading blinded evaluation. The sum of these two cases in 22 post-treatment determinations was 27% (6/22). Furthermore, researchers propose several strategies for implementing blinded evaluations in clinical trials after this trial, which encompass aspects such as staff allocation, training, participant management, trial drug administration, efficacy indicator collection, and safety event management.

**Conclusions:**

The study highlighted that evaluations from non-blinded site investigators may potentially exaggerate the efficacy of the experimental group and that deep wounds can present challenges for observation via center-reading photos. These findings underline the vital necessity for objective assessment in open clinical trials, especially those where wound observation serves as the primary efficacy indicator. The study suggests the adoption of independent blinded investigators at each site, complemented by a comprehensive set of standard operating procedures for blinding evaluation. These measures could serve as an effective counterbalance to subjective bias, thereby augmenting the credibility and consistency of results in open clinical trials. The implications of these findings and recommendations could be of great significance for the design and execution of future open clinical trials, potentially bolstering the quality of clinical research in this area.

**Trial registration:**

ChiCTR2000041443. Registered on December 2020

**Supplementary Information:**

The online version contains supplementary material available at 10.1186/s13063-023-07652-y.

## Introduction

The application of the most reliable evidence to address individual clinical issues underpins the core principle of evidence-based medicine [1]. The rigorous management of trial biases and safeguarding the internal consistency of trials are fundamental to acquiring high-level evidence. Randomized controlled clinical trials (RCTs), often referred to as the “gold standard,” have been employed extensively to blind participants and investigators, thus eliminating potential non-pharmacological influences. Studies have shown that an unblinded design can inflate the perceived treatment effect by 27% to 68% and may even precipitate contradictory conclusions [2–4]. Consequently, the use of blinded designs in trials is deemed necessary.

However, due to the particularity of certain interventions, it becomes challenging to blind participants and investigators in some clinical trials. Recent investigations have highlighted that traditional Chinese medicine (TCM) clinical trials encounter significant obstacles in placebo production, rendering some medications unsuitable for double-blind controlled trials [5, 6]. In response to these challenges, an increasing number of open clinical trials have adopted blinded evaluation methods in recent years [7–11]. However, our preliminary research suggests a dearth of relevant literature concerning the application of blind evaluation.

In this study, we use an open randomized controlled trial of TCM as an example to compare the outcomes of unblinded and blinded evaluations. Furthermore, we propose several strategies for implementing blinded evaluations in clinical trials, which encompass aspects such as staff allocation, training, participant management, trial drug administration, efficacy indicator collection, and safety event management.

## Methods

### Designs

A multicenter RCT on diabetic foot infections (DFIs) was taken as an example. This trial evaluated the efficacy and safety of a Chinese patent medicine. Patients with DFI, aged between 18 and 75 years, and IDSA/IWGDF grade 2 were included [12]. The degree of diabetic foot infection gradually increased from grade 1 to grade 4 according to the IDSA/IWGDF criterion. The total sample size was 240 cases. The patients were treated once a day for two weeks and evaluation indexes were collected weekly. The primary evaluation indicators were infection control rate and infection control time. The secondary evaluation indicators include wound healing rate, bacterial count, amputation rate, the TCM pattern quantification scale for diabetic foot, and wound healing time.

There is a greater risk of using a placebo control for patients in this trial. Furthermore, placebos are challenging to match the investigational drug in terms of color, texture, and odor [5]. Experienced physicians can easily discern information about the group of interventions through the drug exchange process. This study could not implement a double-blind.

To ensure the objectivity and consistency of the test results, using objective evaluation methods to evaluate the efficacy indicators is necessary. We envisioned a blinded evaluation of efficacy indicators using a center-reading blinded method. The center-reading blinded method refers to using a digital camera to take pictures of the condition of the subject’s foot and using the image analysis software ImageJ to analyze the trauma area. We compared the differences between unblinded and center-reading blinded evaluations to investigate whether this method better reflects an accurate picture of efficacy. In addition, other perspectives were collected at the end of this study, including with those involved in designing and conducting the recent blinded evaluation trial.

### Setting

This was an exploratory study, and we randomly selected only about 10% of the completed clinical trial subjects for blinded and unblinded evaluations (*N*=16, *N*experimental group VS *N*control group = 1:1). The study evaluators were divided into non-blinded and blinded investigators who performed the evaluation were associate chief or chief vascular surgeons. And the blinded investigators consisted of three clinicians who were not involved in the clinical study. All unblinded and blinded investigators in this study received training in IDSA/IWGDF grading.

#### Non-blinded investigators

The IDSA/IWGDF grading of DFI was determined and recorded by a non-blinded investigator at the study center for post-treatment patients. A note was made as to whether the subject had local pressure or pain, local fever, systemic symptoms, or symptoms of infection at the time of photography. For the randomly selected subjects, the results of IDSA/IWGDF grading and notes will be filled in the “Study Center Grading Results Evaluation Form” (Appendix 1). Then, the non-blinded investigator desensitized the pictures and sent the messages to the blinded center-reading investigators.

#### Blinded investigators

Based on the “Study Center Grading Results Evaluation Form,” the center-reading investigators made independent determinations back-to-back in a blind state. A third center-reading investigator made a second determination if two center-reading investigators made inconsistent determinations.

### Evaluation

IDSA/IWGDF grading: Assessing the IDSA/IWGDF grading of non-blinded and blinded investigators.

### Quality control and assurance

Previously, we provided better training to the investigators involved in this study’s blinded and unblinded clinical assessment. We believe that the main source of the difference between blinded and unblinded researchers’ evaluation of post-treatment outcomes is evaluation bias, which is the effect of unblinded personnel’s knowledge of the grouping on the evaluation results. For quality improvement methods, data analysis, and reporting, Standards for Quality Improvement Reporting Excellence (SQUIRE 2.0) were referenced [13].

### Statistical analysis

An independent statistician will carry out statistical analysis using the SPSS 25 software. All statistical tests will be two-sided, *P* ≤ 0.05 will be regarded as significant. A 95% confidence interval will be utilized. The Kappa consistency test will assess the results of the non-blind and blind evaluations, and the inferential statistics (*P*-value) will be displayed as descriptive results.

## Results

### Results for blinded evaluation

**Fig. 1:**
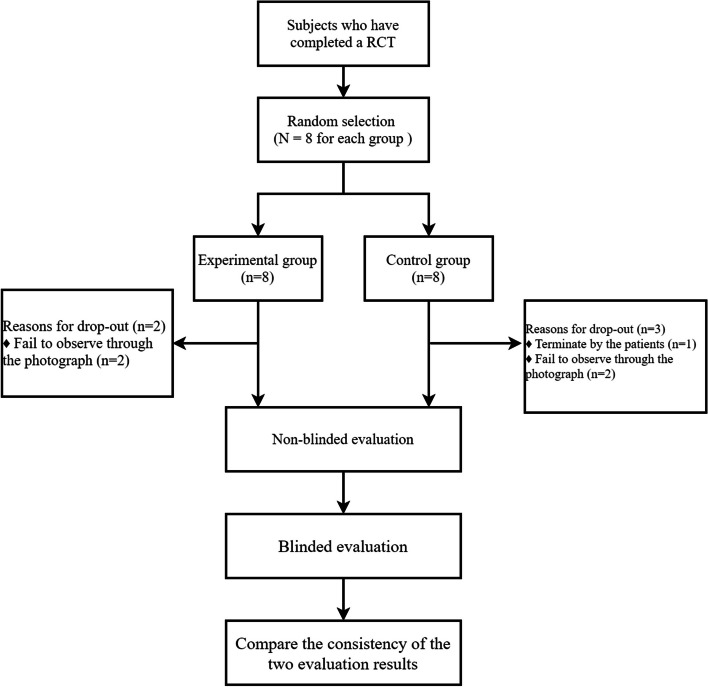
Flow chart for subject

Among the 16 randomly enrolled patients, one was excluded due to a lack of post-treatment follow-ups, and 4 were unable to be analyzed as the central reader could not discern the deeper parts of the wounds from the photographs. The randomization and follow-up process for patients is illustrated in Fig. 1.

The post-treatment IDSA/IWGDF gradings were compared across 11 subjects, revealing inconsistencies between non-blinded and center-reading blinded evaluations according to the consistency test (Kappa=0.248, *P*=0.384). Subsequent comparisons of post-treatment IDSA/IWGDF gradings were carried out within the experimental and control groups separately.

Within the experimental group, three cases were deemed grade 1 by the non-blinded evaluation and were rated grade 2 by the center-reading blinded evaluation. Inconsistencies were also observed within this group between non-blinded and blinded evaluations (Kappa=0.273, *P*=0.523).

In the control group, three cases considered grade 2 in the non-blinded evaluation were rated as grade 1 in the center-reading blinded evaluation. Here too, inconsistencies between non-blinded and blinded evaluations were detected (Kappa=0.273, *P*=0.571). Detailed information is provided in Tables 1, 2, and 3.

**Table 1:** Comparison of IDSA/IWGDF grading results after treatment

		Center-reading blinded evaluation		
		Grade 1	Grade 2	Total
Non-blind evaluation				
	Grade 1	5	4	9
	Grade 2	4	9	13
Total		9	13	22

**Table 2:** Comparison of IDSA/IWGDF assessment results after treatment in the trial group

		Center-reading blinded evaluation		
		Grade 1	Grade 2	Total
Non-blind evaluation				
	Grade 1	2	3	5
	Grade 2	1	6	7
Total		3	9	12

**Table 3:** Comparison of IDSA/IWGDF assessment results after treatment in the control group

		Center-reading blinded evaluation		
		Grade 1	Grade 2	Total
Non-blind evaluation				
	Grade 1	3	1	4
	Grade 2	3	3	6
Total		6	4	10

### Considerations for blinded evaluation

In open clinical trials where wound observation serves as the primary efficacy measure, we recommend the appointment of an independent blinded investigator at each trial site, accompanied by the implementation of a series of standard operating procedures (SOPs) for blinded evaluation. The ensuing considerations for blinded evaluation are explored from three perspectives: safeguarding participants' rights, minimizing bias, and optimizing clinical operability.

#### Personnel roles and training

We advise establishing clearly delineated roles among the research team, which may include treatment investigators, blinded evaluation investigators, research nurses, quality control officers, medication administrators, and documentation managers.

The investigator responsible for efficacy evaluation remains blinded throughout the study, while all other personnel are unblinded. Staff roles should be defined and authorized prior to the trial (refer to Table 4 for example). Comprehensive training should also be provided in multiple sessions such as investigator meetings and study center kickoff meetings. Key training content should encompass the principles and importance of blinded evaluation, operational procedures for conducting a blinded evaluation, risk factors associated with blinded evaluation, communication between non-blinded and blinded investigators, process documentation, and file management. Additionally, all investigators should sign a blinded maintenance pledge to emphasize the significance of implementing blinded evaluations. Should new investigators join the trial during its course, they must receive timely training, and sign the blinded maintenance commitment letter.

**Table 4:** List of responsibilities of principal investigators

Role	Responsibility
Drug administrator	
	1. Receipt of medication into storage and maintenance; 2. Distribution and recovery of drugs, medication compliance records.
Treatment researcher	
	1. Requesting randomization and medication numbers for patients on the central randomization system; 2. Prescribing. 3. Wound clearing and dressing changes were performed on the subjects
Research nurse	
	1. Pick-up medication; 2. Assisting the treatment investigator with wound cleaning and medication change operations.
Blind evaluation researcher	
	1. Screening of subjects; 2. Observation and evaluation of the efficacy index and safety index.
Quality control officer	
	1. Develop a quality control plan; 2. Audit to maintain a blind state of operating procedures; 3. Implement quality control according to the quality control plan.
Document manager	
	1. Preparation, organization, and filing of all documents; 2. Manage and save files separately by blind and non-blind types.

#### Management of participants

Given the external nature of the treatment employed in this study, achieving absolute blinding of participants was challenging. First, both treatment investigators and research nurses should be strictly instructed not to reveal specific treatment protocol details to patients. Secondly, to minimize the potential for inter-patient communication, it is advisable to avoid accommodating patients from different treatment groups in the same room. Lastly, patients must receive thorough education and it must be emphasized that they should not share their treatment information with anyone except their treating physician and research nurse. The participants’ understanding of these crucial blinding maintenance requirements will be assessed during the participant screening process. Any participant unable to comprehend these requirements will not be included in the study.

#### Management of experimental medications

It is recommended that a medication manager be appointed within the study team, responsible for the receipt, storage, maintenance, distribution, and retrieval of medications. The medications should be stored in opaque medicine cabinets. Prior to prescribing, the treatment investigator must obtain a randomization number and drug number from the central randomization system, following which the study nurse is instructed to retrieve the medication from the medication manager. During this process, the medication manager should ensure neither the blinded study personnel nor patients are present. The study nurse collects the medication and directly transfers it to the treatment investigator, taking care to avoid the presence of blinded center-reading investigators. If for any reason the medication cannot be immediately utilized following collection, it should be temporarily stored in areas inaccessible to blinded personnel. In the event a participant withdraws from the trial, the study nurse should promptly return the unused medication to the medication manager.

#### Collection of efficacy indicators

Though the wounds in this study were documented using digital photography and analyzed with image analysis software, factors such as environment, operational procedure, and patient positioning may influence the quality of the images. As such, a blinded assessment investigator should be assigned to document wound healing rates. Additionally, within this study, blinded investigators at the research center should determine the infection control rate, the score for quantitative TCM evidence, and the degree of epithelialization in wound healing. Prior to collecting these indicators, it is recommended that the following process and division of labor be adhered to (i) the treatment investigator and study nurse should remove all dressings, document any purulent discharge from the wound, and perform routine disinfection and cleaning of the wound; (ii) the study nurse should then notify the blinded evaluation investigator; and (iii) the blinded evaluator should proceed with the efficacy evaluation and capture the necessary photographs.

#### Management of safety events

The responsibility for collecting and assessing safety events in this study fell to the blinded investigator, who made determinations of causality based on the five principles of adverse event-drug causality assessment [14]. In the event of serious adverse events, subjects should be promptly treated and the events reported to the members of the Data Monitoring Committee (DMC) within the required timeframe as stipulated by the DMC’s constitution. Should it be necessary to know the intervention group to provide treatment due to safety considerations, this should be done by a non-blinded investigator. If, in an emergency, a blinded investigator learns of a subject’s grouping while participating in emergency treatment, protocol deviations should be documented and brought up for discussion at a blinded review meeting.

## Discussion

In the present study, there were two independent evaluations of each subject at different time points of the visit after treatment, which can be considered a sample size of 22. If we assume that Kappa=0.7 represents a better agreement, while we evaluated the blinded and non-blinded state, we found Kappa=0.248 (about 0.25). With α=0.05, proportions=0.4, 0.6, and according to the above parameters, we used PASS 22 to calculate power=0.75. The findings from our study suggest that even with a relatively small sample size, the outcomes are notably indicative of similar future investigations.

The current study showed that the post-treatment IDSA/IWGDF grading results were compared in 11 subjects, and the consistency test showed inconsistent results between the non-blinded and center reading blinded evaluations (Kappa=0.248, *p*=0.384). In the experimental group, three cases were judged as grade 1 in the non-blinded evaluation and grade 2 in the central reading blinded evaluation; in the control group, three cases were judged as grade 2 in the non-blinded evaluation and grade 1 in the central reading blinded evaluation. The sum of these two cases in 22 post-treatment determinations was 27% (6/22), and the results suggest that the non-blinded determination may have a subjective bias to exaggerate the efficacy of the test group. This is consistent with the results reported in several previous studies [2–4].

While double-blinding is a pivotal measure to control subjective bias from investigators and subjects, it is equally crucial to establish independent blinded evaluations of investigators in scenarios where medications cannot be completely blinded. Even in double-blind trials, independent blinded evaluations are necessary to avoid investigators or patients guessing about intervention groupings by comparing study drugs or based on post-dose responses. However, a research study showed that blinded evaluations were not adequately used and reported, while open trials did not use independent evaluators more frequently than double-blind trials [15].

One study addressed the issue of bias in determining the results of an open randomized controlled trial by administering a questionnaire to the evaluators at the end of the trial [16]. The questionnaire included guesses about whether they thought the subjects belonged to the intervention or control group, the degree of certainty about the answers, the items on which the guesses were based, and whether information about the procedure had been inadvertently revealed. The questionnaire provides a quick insight into the success of the blinded evaluation of this study, while facilitating statistical analysts to estimate and eliminate confounding factors affecting the determination of outcomes based on a causal inference framework to obtain unbiased estimates of efficacy and more easily interpretable estimates.

The implementation of blinded evaluation is a significant challenge for the design and implementation of the study. In open clinical trials where wound observation is the primary indicator of efficacy, it is recommended that there be an independent blinded investigator at each trial site, and a series of standard operating procedures (SOPs) for blinded evaluation should be implemented. We have described above the basic requirements for blinded evaluations by setting up standard processes: separation of duties for investigators, subject management, collection of efficacy and safety indicators, and management of trial drugs. In some studies, when scientificity and operability permit, we propose that blinded evaluation can be conducted by taking photos and centralized evaluation by an independent evaluation committee to ensure the objectivity and consistency of the results. For some of the objective result data (e.g., essential test data, metabolomics data) for which there is a risk of blinding, we suggest that blinded management can be used, where the personnel blinded to the evaluation of efficacy and safety indicators are not exposed to these data results. Meanwhile, research team members at the back of the data chain (e.g., data management, data statistics) should also be in a blinded state to reduce bias. Research quality control personnel should follow the PDCA cycle model (e.g., planning, doing, checking, and acting) for all phases of quality management-related work. Through the above process, ensure the maintenance of blinded evaluation throughout the trial and improve the quality of open clinical trials.

Although we have proposed various measures to mitigate bias in open clinical trials, further exploration and innovation are necessary to enhance the reliability of trial outcomes. For instance, introducing objective efficacy evaluation tools, like sensor-equipped insoles, can provide unbiased, quantifiable data [17]. In addition, strengthening the blinded management throughout the entire data chain, from data collection to statistical analysis, can also help reduce bias.

Despite these strengths, our study is not without limitations. The relatively small sample size may raise questions about the reliability of the results. Future research should validate our findings with larger sample sizes and further explore the best practices for maintaining blinded evaluation in clinical trials.

## Conclusions

In open clinical studies, minimizing information bias represents a significant challenge. Our findings indicate that implementing blinded evaluations can effectively reduce information bias, thereby ensuring the authenticity and reliability of trial outcomes. We strongly advocate for the appointment of an independent blinded researcher at each trial site and the implementation of a series of standard operating procedures (SOPs) for blinded evaluations. For this purpose, we propose a comprehensive set of SOPs encompassing divisions of labor among researchers, subject management, collection of efficacy and safety indices, and trial drug management. By doing so, we aim to achieve effective blinded evaluations and enhance the quality of open trials to the greatest extent possible.

Importantly, our study provides a practical strategy for future clinical trials to mitigate potential biases, thereby strengthening the evidence base for clinical decision-making.

### Supplementary Information

**Additional file 1.** Study Center Grading Results Evaluation Form.

## Data Availability

The datasets will be made available after a reasonable request to the investigator of the Institute of Clinical Pharmacology, Xiyuan Hospital, Chinese Academy of Traditional Chinese Medicine.

## Abbreviations

TCM: Traditional Chinese medicine
DFI: Diabetic foot infection
RCT: Randomized controlled clinical trial
SOPs: Standard operating procedures
