# Supplementary material for: Enhancing open clinical trials through blinded evaluations: an exploration with diabetic foot infections
Source: Trials. 2023 Nov 9;24:716. doi: 10.1186/s13063-023-07652-y (PMC10636892; doi:10.1186/s13063-023-07652-y)
Supplement: Supplementary file 1 — Additional file 1. Study Center Grading Results Evaluation Form. [file 13063_2023_7652_MOESM1_ESM.docx]

**Appendix 1**

**Study Center Grading Results Evaluation Form**

| **Center number** | **Random number** | **Random time** | **Time of picture taking** | **Center-reading investigator's judgment grading** | **Notes from the center-reading investigators** | **Notes from the non-blinded investigators** | **Situation of dropout** |
| --- | --- | --- | --- | --- | --- | --- | --- |
| 01 | R002 | 2021-03-10 |  |  |  |  | No |
|  |  |  |  |  |  |  |  |
|  |  |  |  |  |  |  |  |
| 01 | R004 | 2021-03-10 |  |  |  |  | No |
|  |  |  |  |  |  |  |  |
|  |  |  |  |  |  |  |  |
| 01 | R020 | 2021-07-29 |  |  |  |  | No |
|  |  |  |  |  |  |  |  |
|  |  |  |  |  |  |  |  |
| 01 | R118 | 2021-10-22 |  |  |  |  | No |
|  |  |  |  |  |  |  |  |
|  |  |  |  |  |  |  |  |
| 02 | R028 | 2021-08-02 |  |  |  |  | No |
|  |  |  |  |  |  |  |  |
|  |  |  |  |  |  |  |  |
| 02 | R031 | 2021-08-02 |  |  |  |  | No |
|  |  |  |  |  |  |  |  |
|  |  |  |  |  |  |  |  |
| 02 | R044 | 2021-08-07 |  |  |  |  | No |
|  |  |  |  |  |  |  |  |
|  |  |  |  |  |  |  |  |
| 02 | R080 | 2021-08-24 |  |  |  |  | No |
|  |  |  |  |  |  |  |  |
|  |  |  |  |  |  |  |  |
| 02 | R100 | 2021-09-04 |  |  |  |  | Yes |
| 02 | R101 | 2021-09-09 |  |  |  |  | No |
|  |  |  |  |  |  |  |  |
|  |  |  |  |  |  |  |  |
| 03 | R073 | 2021-08-19 |  |  |  |  | No |
|  |  |  |  |  |  |  |  |
|  |  |  |  |  |  |  |  |
| 03 | R105 | 2021-09-22 |  |  |  |  | No |
|  |  |  |  |  |  |  |  |
|  |  |  |  |  |  |  |  |
| 04 | R027 | 2021-07-31 |  |  |  |  | No |
|  |  |  |  |  |  |  |  |
| 04 | R123 | 2021-11-10 |  |  |  |  | No |
|  |  |  |  |  |  |  |  |
| 05 | R090 | 2021-09-01 |  |  |  |  | No |
|  |  |  |  |  |  |  |  |
|  |  |  |  |  |  |  |  |
| 05 | R128 | 2021-11-10 |  |  |  |  | No |
|  |  |  |  |  |  |  |  |
|  |  |  |  |  |  |  |  |

**Notes:**

**Non-blinded investigators:** You should observe the subject's foot and complete "Notes from the non-blinded investigators" based on whether the subject had local pressure or pain, local fever, systemic symptoms, or symptoms of infection.

**Blinded investigators:** You should analyze the images in conjunction with the "Notes from the non-blinded investigators" . After careful review, please complete the corresponding sections based on the IDSA grading: "Center-reading investigator's judgment grading" and "Notes from the center-reading investigators" .
